# Organizational Commitment Profiles and Turnover Intention: Using a Person-Centered Approach in the Korean Context

**DOI:** 10.3389/fpsyg.2019.01499

**Published:** 2019-07-03

**Authors:** Hyun Sung Oh

**Affiliations:** School of Business Administration, Chonbuk National University, Jeonju, South Korea

**Keywords:** organizational commitment, latent profiles, person-centered, mixture model, turnover intention

## Abstract

Extensive research on organizational commitment (OC) profiles has been conducted as researchers recognized that individuals can concurrently experience various levels of OC. Although there is a growing body of research on commitment profiles (CPs) within the past decade, existing empirical research has focused on Western cultural contexts and paid little attention to CPs arising in Asian cultural contexts. The present study utilized a person-centered analytic method (latent profile analysis) to examine the types of CPs among a sample of employees from South Korea (*n* = 510). From the results emerged six distinct CPs of South Korean employees, and these six profiles exemplified the differing impact of CPs on turnover intentions. Finally, the implications of these results for commitment theory, practice, and future research are discussed.

## Introduction

Research on the relationship between organizational commitment (OC) and turnover intention (TIN) (Porter et al., 1974; Mathieu and Zajac, 1990) has a long history, with Meyer and Allen’s three component model (Allen and Meyer, 1990) arguably being the most dominant model of OC. Meyer and Allen proposed that commitment is multi-dimensional and consists of three components: affective, continuance, and normative commitment (NC). Affective commitment (AC) refers to an employee’s emotional attachment to the organization, continuance commitment (CC) refers to the need to remain with the organization due to the perceived cost associated with leaving the organization, and NC refers to an employee’s sense of obligation to remain with the organization (Allen and Meyer, 1990, 1996; Meyer and Allen, 1991). Meyer et al.’s (2002) meta-analysis of 155 studies of OC found that TIN was negatively correlated with the AC, CC, and NC. The meta-analysis, which was reaffirmed by another meta-analysis conducted by Meyer et al. (2012a) a decade later, indicated variations in the strength of the relationship between TIN and each of the components of OC, the strongest relationship being AC.

Studies on OC have tended to focus on the components of commitment as individual variables (Stanley et al., 2013). This is despite Allen and Meyer’s (1990) earlier proposition that each component of OC may simultaneously be experienced at varying degrees, and furthermore, that these configurations call for closer investigation as they will have different implications for work behaviors and intentions (Oh, 2016). Their proposition implied that the three components of commitment generate an employee’s mindset and should thus be considered as components within a commitment profile (CP). Meyer and Herscovitch (2001) argued that an employee’s commitment is a “mindset” which manifests in various forms such as employee desire (i.e., AC), perceived cost (i.e., CC) or obligation to (i.e., NC) continuance as a cause of action. That is, employees can experience different levels of each of the three components simultaneously, a proposition first put forward by Allen and Meyer (1990).

Several recent empirical studies on (e.g., Wasti, 2005; Gellatly et al., 2006; Meyer et al., 2012b, 2013a; Stanley et al., 2013; Kam et al., 2016) have argued that the different commitment dimensions interact to create distinct profiles of commitment, which have differing employee consequences such as employee TIN and organizational citizenship behavior (OCB). Despite the increased theoretical interest in CPs, research on CPs has mostly been conducted in Western countries, while research on CPs in non-Western countries remain sparse. The purpose of the current study therefore, is not only to utilize a person-centered approach to identify employees’ distinct patterns of CPs based on the combinations of the commitment components, but also to examine how the CPs of employees in a non-Western context are associated with employees’ work-related behavior such as TIN. The following section will review the literature on OC, clarifying the differences between a person-centered and a variable-centered approach with respect to the identification of CPs.

### Identifying Commitment Profiles: Variable-Centered vs. Person-Centered Approaches

While extensive research has been conducted on the three-component model of OC, prior studies have tended to utilize a variable-centered approach through the use of regression/correlation analysis (see Meyer et al., 2002). In recent years, an emerging stream of OC research has shifted from a variable-centered approach to a person-centered approach to examine the different configurations of the commitment components (e.g., Somers, 2010; Meyer et al., 2012b, 2015; Kam et al., 2016).

Using a variable-centered approach has provide better understanding OC by testing independent and interaction effects, and by identifying different types of commitment. However, moderated multiple regression using a variable-centered approach, while it can distinguish interactions among the commitment components, cannot identify the groups within a sample (Oh, 2016). Therefore, variable-centered approaches overlook the possibility “that the participants may come from different subpopulations in which the observed relations between variables may differ qualitatively and quantitatively” (Morin et al., 2011, p. 59). Moreover, such an approach to studying OC has recently been criticized as inadequate for distinguishing complex interactions as the combinations of forms of commitment to multiple facets are too intricate (Meyer and Morin, 2016; Morin et al., 2016). In contrast, a person-centered approach enables insight into how types of commitment may combine and how they may be experienced simultaneously by employees, in addition to how groups fitting various CPs may be different in terms of other variables (Meyer et al., 2015). By identifying different configurations researchers are thus able to confirm varying implications for employee work-related behaviors such as TIN (Meyer et al., 2012b, 2015; Stanley et al., 2013). Hence, there are evident advantages to taking a person-centered approach. The following section, will review research on CPs utilizing a person-centered approach.

To identify the pattern of CPs, early research utilized a median split analysis. Median split involves the dichotomization of a continuous independent variable into a categorical variable having two groups. Such simplification of the statistical analysis allows greater ease of interpretation and presentation of results (MacCallum et al., 2002; Meyer et al., 2013a, 2015). However, several researchers have asserted that median split analysis cannot answer the question of whether these profiles are naturally created within a sample, as the median is an arbitrary threshold (Pastor et al., 2007; Meyer and Morin, 2016; Morin et al., 2016). For this reason it would be beneficial to use an analytic method such as cluster and latent profile analysis (LPA) that detects natural groupings rather than arbitrary ones. By identifying natural groups, cluster analysis performs partitioning of the data (MacCallum et al., 2002). It also provides the conceptual structure of the data by categorizing discrete types or group of individuals that combine and form the most similar or dissimilar observations within a data set (Aldenderfer and Blashfield, 1984; Oh, 2016). However, the reliability of the results of cluster analysis may be called into question as the researcher can subjectively produce a cluster of equal size (Meyer et al., 2012b; Morin et al., 2016). In order to overcome this advanced studies on CPs have utilized LPA. Vandenberg and Stanley (2009) argued that adopting LPA for the study of CPs could allow examination of the dynamic, synergistic effects of commitment. LPA allows researchers to compare alternative profile models and identify the most appropriate one through the evaluation of the relative model fit statistics. LPA identifies subgroups of the population having similar patterns of scores on the set of commitment variables (Morin et al., 2011; Meyer et al., 2012b). The above-mentioned points, including arbitrariness associated with the dichotomization of a continuous variable and identification of inherent groupings in data as recognized by median split or cluster analysis clearly indicate that LPA is a superior statistical technique. For these reasons, in this study LPA was selected for the identification of CPs.

### Commitment Profiles

Previous research on OC demonstrated that AC and NC are positively related to employee work-related outcomes, while CC is negatively related (Meyer et al., 2002). There have been five to seven CPs with distinct patterns of AC, NC, and CC revealed across a number of studies (e.g., Meyer et al., 2012b, 2015; Stanley et al., 2013). The consistent pattern of CPs from the previous studies includes some version of AC-dominant, CC-dominant, AC-NC-dominant, fully committed, and uncommitted. In addition, some studies have identified moderate CP groups with moderate levels of all three components (Wasti, 2005; Meyer et al., 2012b, 2015; Stanley et al., 2013; Oh, 2016). For example, Wasti’s study categorized employees displaying levels of AC or CC that were above average as AC-dominant or CC-dominant, while referring to employees who scored slightly less than average on all commitment forms as “Neutrals.” The term “Moderate Commitment” was used in Meyer et al. (2012b) study regarding CPs that were closest to the mean levels. As indicated by these studies, parts of distinct patterns of CPs should include moderate levels of AC, NC, and CC. Notably, a widely accepted cut-off point between high and moderate levels of AC, NC, and CC, has not been defined. Extant research on CPs has used either relative levels (based on cluster and LPA) or above/below median scores (median split analysis) to label profile groups such as AC-dominant (Oh, 2016). Based on Meyer and Hersovitch’s notion of CPs, it is commonly found that employees most likely to remain with their organization fit CPs relating to strong AC and weak NC and CC.

Previous studies (Wasti, 2005; Gellatly et al., 2006) on CPs recommended further exploration of the complex mechanism existing in the coexistence of the three components of commitment in order to gain a more complete understanding of CPs. For instance, Gellatly et al. (2006) argued that the role of NC may be dependent on the relative levels of AC and CC. Further, they argued that the notion of a context effect may be helpful to explain how the role of NC changes based on the relative levels of AC and CC. The following section will discuss the context effect on CPs.

### Context Effect Within Commitment Profiles

According to Gellatly et al. (2006), there is the potential for a component within the CP to be affected by the strengths of the other components that are present. They asserted that the relative strengths of AC and CC within an employee’s CP can provide a context that will influence the specific nature of NC, causing it to emerge as either indebted obligation or moral imperative. With regards to the nature of NC, conceptual studies have not made clear predictions for combinations including those, where levels of CC or AC are moderate. Instead, such studied have only extended to CPs, where CC or AC are high, i.e., low AC and high CC, or high AC and low CC. Accordingly, further investigation into CPs could potentially allow new insights into the nature of NC when the levels of both AC and CC are high or moderate within CPs (Oh, 2016).

A more complex conception of AC–CC interaction could provide the basis for predictions regarding the nature of NC. Rather than merely distinguishing between high and low levels of these two components, the present study proposes that a continuum of levels should be considered when determining their combined effect on NC. In fact, regarding “context effect” in NC, the effect of AC may be stronger compared to CC (Meyer and Herscovitch, 2001; Gellatly et al., 2006; Meyer and Parfyonova, 2010; Meyer et al., 2012b; Stanley et al., 2013; Oh, 2016). Consequently, moral imperative could be associated even with profiles when AC and CC are moderate, and not only when both components are high. However, when AC is moderate and CC is low, “moral imperative” seems inadequate to describe the nature of NC, as it generally consists of positive beliefs and affect toward the organization (i.e., internally driven). Similarly, in the case of low AC and moderate CC, the nature of NC may resemble indebted obligation as CC may be more dominant due to the low level of AC. However, generally the nature of NC as indebted obligation consists of less positive beliefs and commitment (i.e., weakly internally driven) toward the organization. As such, the term “indebted obligation” seems insufficient to describe the nature of NC when AC is low and CC is moderate (Oh, 2016).

### Cultural Context Effect on Turnover Intention Within Commitment Profiles

All three-commitment components are theorized to be associated with TIN (Meyer and Herscovitch, 2001). However, the causal mechanism underlying each component is different (Allen and Meyer, 1990; Meyer and Allen, 1997; Meyer and Herscovitch, 2001). Studies discussed here have considered internal and external factors influencing commitment components, but with a Western cultural context. These studies found that employees with a high level of AC, who are emotionally committed, have a strong desire to remain in the organization – compared to those who are low in AC (Meyer et al., 2002; Stanley et al., 2013). This is because employees’ AC is associated with positive beliefs and affect (internal drive) toward their organization (Meyer et al., 2012b; Stanley et al., 2013; Meyer and Morin, 2016). Individuals with strong NC can feel a similar level of intention to stay, motivated by a sense of obligation to the organization or a desire to reciprocate they gain from the organization (Meyer et al., 2002; Meyer and Morin, 2016). Employees with strong CC, who recognize that high costs will be associated with leaving the organization, are more likely to remain with the organization because they do not want to lose the benefit of organizational membership or because they perceive the lack of alternatives (Meyer and Morin, 2016).

Stanley et al. (2013) argued that the salience of both internal and external drive is closely related to employees voluntarily leaving their organization. They argued that AC plays the role of internal drive for employee TIN because it is based on internal reasons such as positive beliefs and affect toward the organization. CC then plays the role of external drive for employee TIN as it is associated with external, rather than internal, reasons. The majority of research on CPs has demonstrated that profiles with high AC (e.g., AC-dominant, AC–NC dominant, fully committed) are associated with a low level of employees’ TIN. On the other hand, profiles with high CC (e.g., CC-dominant) are associated with a high level of employees’ TIN. As discussed above, the effect of both internal drive and external drive on employee TIN is dependent on the strengths of AC and CC within CPs in terms of within-person context (Stanley et al., 2013). This argument initially stems from Gellatly et al.’s (2006) position regarding the dual nature of NC depending on the relative level of AC and CC within CPs.

The studies mentioned above have been carried out in a Western cultural context, which can be characterized as individualist, while non-Western cultures can be roughly characterized as collectivist (Wasti, 2005; Fischer and Mansell, 2009; Morin et al., 2015; Meyer and Morin, 2016). The differences between these cultural contexts may have a significant impact on the nature of the constitution of employees’ CPs. Despite the potential heterogeneity in CPs depending on cultural values, there is still a lack of understanding about CPs in non-Western cultures, compared to those in Western cultures (Fischer and Mansell, 2009; Morin et al., 2015; Meyer and Morin, 2016). For example, Bergman (2006) argued that the norm-oriented approach of being dutiful and fulfilling obligations to others is resented by those in an individualist culture, while being pleasing to members of a collectivist culture. Empirical research carried out in collectivist cultural contexts has found that employees in collectivist societies tend to remain with an organization and put discretionary effort into their work due to a sense of societal obligation, more so than those in individualistic contexts (Fischer and Mansell, 2009; Meyer et al., 2012a; Meyer and Morin, 2016). It is worthwhile to investigate not only the patterns of CPs based on the combination of AC, NC, and CC but the relationships between CPs and employee work-related behavior such as TIN in non-Western culture. Taken altogether, the following hypotheses are offered.

H1: The combination of AC, NC, and CC will reveal multiple (five to seven) profile groups including moderate levels of AC, NC, and CC within the employee sample.
H2: Profile groups with moderate to strong NC in combination with strong AC (e.g., AC/NC-dominant and fully committed) will have lower levels of TIN and then profile groups with moderate to strong NC but weak AC (e.g., NC-dominant, CC/NC-dominant).
H3: Profile groups with moderate to strong NC in combination with relatively strong CC compared to AC (e.g., CC/NC dominant) will have higher levels of TIN than profile groups with strong AC, but lower levels of TIN than profile groups with low levels of NC and AC (e.g., CC-dominant, uncommitted).

## Materials and Methods

The data for the present study were drawn from employees of randomly selected companies in South Korea. A total of 600 pencil-and-paper surveys were distributed and 517 surveys were collected. Incomplete surveys were excluded from the collected data for statistical analysis. This left 510 returned usable surveys remaining for a response rate of 85%. A sample of 510 Korean employees was recruited from six different industry sectors: 117 (22.9%) from the manufacturing sector, 30 (5.9%) from the sales sector, 252 (49.4%) from the general management sector, 56 (11%) from the service sector, and 55 (10.8%) from the research sector. Among the participants, 298 (58.4%) were male and 212 (41.6%) were female. Of the participants, 219 (42.9%) were unmarried and 291 (57.1%) were married. The ages of participants ranged from those in their 20 s at 18.8%; 30 s at 38.4%; 40 s, 23.1%; and 50 s and over, 19.6%. With respect to educational background, 226 (44.3%) had graduated from 4-year university degrees, while 2-year college degrees numbered 148 (29.0%), graduate school and above, 38 (7.5%), and high school graduates numbered 98 (19.2%), respectively.

### Measures

The Korean version of OC measure used in the present study was formerly validated by Lee et al. (2001). Lee et al.’s (2001) 15-item Korean version was originally adapted for international research from Meyer et al.’s (1996) scale created through translation of Allen and Meyer’s (1990) original items into Korean and back-translated into English. Due to the lack of published Korean translation for measures of TIN, the present study utilized Beaton et al.’s (2000) back-translation technique to translate this. In order to make the items equivalent to the original English-language items, adjustments were made such as simplifying the content of the item, abridging items, rephrasing, or eliminating expressions specific to North America ensured that the survey was culturally appropriate and used up-to-date wording. The first author translated the survey from Korean into English, which was subsequently back-translated to English by two Korean academics. Unless otherwise noted, survey items used a 5-point scale ranging from strongly disagree (1) to strongly agree (5).

### Organizational Commitment

As mentioned above, AC, CC, and NC were measured using Lee et al.’s (2001) 15-item Korean translation of an OC scale using a five-point Likert-type response format. An example of an AC item was as follows: “I really feel as if this organization’s problems are my own.” An example of a CC item was “I feel that I have too few options to consider leaving this organization.” An example NC item was “I do not feel any obligation to remain with my current employer.” Previous psychometric evaluation of this measure has demonstrated its reliability and provides a non-overlapping assessment of the three forms of OC (Lee et al., 2001).

### Turnover Intention

Mitchel’s (1981) four-item TIN subscale was used to measure employees’ TIN, with responses being scored on a five-point scale ranging from 1 (strongly disagree) to 5 (strongly agree). The subscale evaluated the likelihood of the respondent leaving the organization in the near future. A sample item was as follows: “I plan to be with the company quite a while.”

### Data Analysis

As a starting point, we first tested the dimensionality of the three-factor commitment measure. A confirmatory factor analysis (CFA) suggested that a three-factor structure fit the data. Item scores for each employee from the CFA were utilized as the indicators for the subsequent LPA. To describe the fit of the model, goodness of fit indices were considered; (a) the comparative fit index (CFI), (b) the root mean square of approximation (RMSEA) and its 90% confidence interval (CI), and (c) the standardized root mean square residual (SRMR). Values > 0.88 for the CFI and TLI indicate acceptable, and values higher than 0.08 and 0.06 for the RMSEA and SRMR, respectively, support adequate and acceptable model fit except for RMSEA (χ2 = 414.619; df = 62; *p* ≤ 0.000; CFI = 0.891; TLI = 0.862; RMSEA = 0.106; CI = 0.096–0.115; SRMR = 0.070).

By utilizing Mplus 7.31, we then conducted the LPA, which identified the pattern of CPs using robust maximum likelihood estimator (MLR) (Muthén and Muthén, 1998–2014). When conducting the LPA, item scores were used as indicators (Meyer et al., 2012b; Stanley et al., 2013). To avoid converging on a local solution (i.e., a false maximum likelihood), we examined all models of 3,000 random sets of start values, 300 iterations, and the 100 best solutions for optimization suggested by Hipp and Bauer (2006) and Morin et al. (2015). We examined separately 1–7 potential profile solutions. In order to choose the optimal number of profiles, we took into account both the substantive meaning and theoretical conformity of the profiles and the statistical adequacy of the solution and a number of statistical indicators such as the Akaike Information Criterion (AIC), the Bayesian Information Criterion (BIC), and the sample-adjusted BIC (SABIC) including entropy. However, researchers such as Lubke and Muthén (2007), Howard et al. (2016), and Morin et al. (2016) postulated that entropy should not be the most important deciding factor used to determine the optimal number of profiles. Overall, a lower value on the AIC, BIC, and SABIC suggest which profile best fits the model. Finally, the retained profiles were compared with outcome (TINs) by using AUXILIARY (BCH) function, which tests the equality of means across profiles as done by Morin et al. (2015). It should be noted that when the retained profiles are compared with outcome variables including covariates, there is the possibility that these variables may influence the nature of the retained profiles. Asparouhouv and Muthén (2014) suggested that AUXILIARY (BCH) would be suitable to address this issue.

## Results

The means, standard deviations, and correlations for the study variables are presented in Table 1.

**Table 1:** Descriptive statistics and correlations among study variables.

	*M*	SD	1	2	3	4
(1) AC	3.52	0.81	(0.88)			
(2) CC	3.00	0.43	0.20^∗∗^	(0.91)		
(3) NC	3.00	0.81	0.60^∗∗^	0.16^∗∗^	(0.75)	
(4) TIN	2.91	0.91	-0.48^∗∗^	-0.09^∗^	-0.25^∗∗^	(0.81)

*n = 510. Internal consistency alphas are in parentheses along the diagonal. AC, affective commitment scale; CC, continuance commitment scale; NC, normative commitment scale; TIN, turnover intention. All values are rounded to two decimal places. ^∗^*p* < 0.05, ^∗∗^*p* < 0.01*.

### Latent Profile Analyses (LPA)

**Figure 1:**
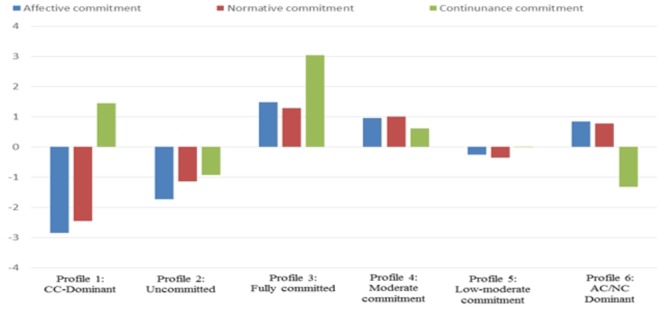
Six-profile solutions identified in this study. Profile indicators are item scores generated from model in which item scores were estimated in standardized units (*M* = 0; SD = l). AC, affective commitment; CC, continuance commitment; NC, normative commitment.

Regarding the aforementioned potential number of profiles, we expected that five to seven latent profiles would be identified; a model including one to seven profiles were estimated. According to the statistical adequacy of the solution and statistical indicators such as AIC, BIC, SABIC, and entropy, a model with six profiles fit the data best. At the same time, we also considered whether the identified patterns of CPs were consistent with the substantive meaning and theoretical conformity of the profiles. As a result, the LPA indicated that a model with six profiles solution fit the data in this study. Table 2 shows various results from the LPA in terms of class enumeration. Table 3 indicates that the number of cases in each of the six profiles ranged from 4 to 306. The posterior probabilities that individuals are the property of their assigned profiles and no other profiles were high (0.73–0.97) (Table 4). Figure 1 shows the patterns of profiles based on the result of LPA.

**Table 2:** Profile enumeration.

	LL	#fp	AIC	BIC	SABIC	BLRT (*p*-value)	LMR (*p*-value)	Entropy
1 profile	-2169.474	6	4350.949	4376.355	4357.311	Na	Na	Na
2 profile	-2077.189	10	4174.377	4216.721	4184.980	0.00	0.00	0.663
3 profile	-2031.598	14	4091.198	4150.480	4106.042	0.00	0.00	0.779
4 profile	-2016.139	18	4068.278	4144.497	4087.363	0.00	0.04	0.718
5 profile	-2001.982	22	4047.964	4141.121	4071.290	0.00	0.09	0.803
6 profile	-1988.413	26	4028.827	4138.921	4056.394	0.00	0.02	0.836
7 profile	-1975.654	30	4011.307	4138.339	4043.115	0.00	0.28	0.851

*LL, log likelihood; #fp, number of free parameters; AIC, akaike information criterion; CAIC, constant AIC; BIC, bayesian information criterion; SABIC, sample size adjusted BIC; BLRT, bootstrap likelihood ratio test; LMR, Lo–Mendell–Rubin likelihood ratio test*.

**Table 3:** Profile membership for the 1-, 2-, 3-, 4-, 5-, and 6-profile models.

Profile	1	2	3	4	5	6
1-profile	510	–	–	–	–	–
2-profile	312	198	–	–	–	–
3-profile	36	169	305	–	–	–
4-profile	36	289	110	75	–	–
5-profile	4	41	51	107	307	–
6-profile	4	35	7	119	306	39

*Indicates the number of cases in each profile for the 1-, 2-, 3-, 4-, 5-, and 6-profile models*.

**Table 4:** Posterior classification probabilities for the most likely latent profile membership (Column) by latent profile (row).

Profile	*n*	1	2	3	4	5	6
1	4	**0.97**	0.019	0.000	0.000	0.002	0.000
2	35	0.000	**0.76**	0.000	0.000	0.234	0.000
3	7	0.000	0.000	**0.86**	0.138	0.000	0.000
4	119	0.000	0.000	0.006	**0.85**	0.111	0.032
5	306	0.000	0.009	0.000	0.040	**0.93**	0.012
6	39	0.000	0.000	0.000	0.109	0.155	**0.73**

*Values in bold are the average posterior probabilities associated with the profiles to which individuals were assigned*.

### Hypothesis Testing

Profiles were labeled as follows: Profile 1 (4 cases or 0.8% of the sample) had greater CC scores than any other profile, but with low AC and NC. This was labeled CC- Dominant. Profile 2 (35 cases or 7% of the sample) had the lowest scores all round (AC low, CC low, NC low), reflecting a general lack of commitment, and was thus labeled Uncommitted. Profile 3 (7 cases or 1% of the sample) reflected overall commitment strength with AC high, CC high, NC high, and was thus labeled Fully committed. Profile 4 (119 cases or 23% of the sample) showed all-round (AC moderate, CC moderate, NC moderate). It was labeled Moderate commitment. Profile 5 (306 cases or 60% of the sample) showed all-round (AC low moderate, NC low moderate, CC low moderate) and was thus labeled Low-moderate commitment. Profile 6 (39 or 8% of the sample) reflected commitment strength with AC moderate, NC moderate and CC low. It was labeled AC/NC-Dominant. The identification of distinct CPs is consistent with previous research (Somers, 2009, 2010; Meyer et al., 2012a; Stanley et al., 2013; Wasti, 2005). These results generally support Hypothesis 1.

Following Morin et al.’s (2015) recommendation, the six profiles solution was contrasted with outcome (i.e., TIN) by using the AUXILIARY (BCH) function to examine statistically mean-level differences across the six profiles. Table 5 presents the level of TINs in the six profiles with a summary of the test statistical significance for the equality of mean levels across the six profiles.

**Table 5:** Characteristics of the profiles of commitment on the outcome.

	Profile 1 CC- dominant	Profile 2 uncommitted	Profile 3 fully committed	Profile 4 moderate commitment	Profile 5 Low- moderate commitment	Profile 6 AC/NC4 Dominant	Summary of significance4 test
Turnoverintention	4.188	4.088	1.363	2.788	2.900	2.325	1 = 2 > 5 = 4 = 6 > 3

According to Hypothesis 2, profile groups with moderate to strong NC in combination with strong AC (e.g., AC/NC-dominant and fully committed) will have lower levels of TIN than profile groups with moderate to strong NC but weak AC (i.e., NC-dominant, CC/NC-dominant). This hypothesis could not be fully tested, as the LPA did not produce NC-Dominant or CC/NC-Dominant profile groups. Nevertheless, as expected, Profile 3 (Fully committed) showed the lowest level of TIN in any other profile group except Profile 2 (Weakly CC-Dominant). In addition, Profile 6 (AC/NC Dominant) showed a significantly lower level of TIN than other profiles (CC-Dominant, Uncommitted). Thus, Hypothesis 2 was supported. According to Hypothesis 3, profile groups with moderate to strong NC in combination with relatively strong CC compared to AC (i.e., CC/NC dominant) will have higher levels of TIN than profile groups with strong AC, but lower levels of TIN than profile groups with low levels of NC and AC (i.e., CC-dominant, uncommitted). Again, because the LPA did not produce the CC/NC-Dominant profile, this hypothesis could be fully tested. However, the results showed that Profile 1 (CC-Dominant) where CC is high but AC and NC are low is associated with the highest level of TIN across the six profiles. As expected, Profile 2 (Uncommitted) also showed a higher level of TIN, but there was no significant difference with Profile 1 (CC-Dominant). Hence, Hypothesis 3 was generally supported. Overall, based on the profile enumeration by the LPA, not all hypotheses could be fully tested. Nevertheless, with respect to TIN, profiles concerning moderate to strong AC and NC showed a lower level of TIN than profiles with a high level of CC (i.e., CC-Dominant).

## Discussion

The purpose of the current study was both to examine the pattern of CPs for participating South Korean employees and to determine whether CPs were predictive of employee’s TIN, as suggested by prior research (Meyer et al., 2013b, 2018; Stanley et al., 2013). This is because CPs based on the combination of AC, CC and NC are associated with the psychological states within organizations that have a bearing on employee work-related behavior. Moreover, as one of the few studies on CPs in a non-Western context (except Wasti, 2005; Morin et al., 2015) his study extended the investigation of CPs utilizing a person-centered approach in the field of OC. By means of LPA, as expected, our findings for a sample of South Korean employees yielded six distinct CPs and supported the idea that employees’ CPs were linked to their TIN. Similar to the findings of previous empirical studies (Meyer et al., 2012b; Stanley et al., 2013; Morin et al., 2015) the results identified six CPs. However, the nature of these profile groups varied quantitatively and qualitatively. That is, this study identified three profile groups that varied across all three components.

As expected, the results indicated that TIN was lowest when AC and NC were high. These findings supported Hypothesis 1 (Gellatly et al., 2006; Meyer and Parfyonova, 2010; Meyer et al., 2012b; Morin et al., 2015). Furthermore, this study further investigated the nature of NC beyond either CC or AC high introduced by Gellatly et al. (2006). At this stage, no other predictions are made for other combinations (e.g., when the levels of CC or AC are moderate). It was also found that employees’ level of TIN was lower when they belonged to profile groups with a moderate to strong level of AC and NC (i.e., Fully committed, AC/NC Dominant, Moderate commitment). Thus it can be speculated that employees’ mindsets associated with these CPs are characterized by moral imperative (Meyer and Parfyonova, 2010), a finding that supports arguments proposed previously that NC is salient in collectivist culture (Wasti, 2003). In other words, the nature of NC may be more likely to be experienced as moral imperative in a collectivist culture as employees feel obligated to reciprocate the benefits received from the organization or to meet other’s expectations (Meyer and Parfyonova, 2010; Meyer and Morin, 2016). Unfortunately, this study did not capture Profile CC/NC Dominant. Even though the level of CC is slightly higher, the level of all three components does not vary greatly, and employees are moderately committed to their organization. This study was therefore not able to test fully the context effects within CPs in terms of indebted obligation. In this study AC-Dominant profile did not appear, in contrast with the majority of research on CPs (Kabins et al., 2016; Morin et al., 2016). Meyer et al. (2012b) argued that the fact that “LPA does not always generate the profile groups needed to test substantive hypotheses is a limitation” (p. 13).

### Theoretical Implications

According to our findings, the Profile 1 (CC-Dominant) showed the highest level of TIN. This might be associated with the operationalization of OC in the context of a different employment sector. Meyer et al. (2018) recently measured different facets of CC by dichotomizing between CC: HS (e.g., “If I decided to leave this organization, too much of my life would be disrupted”) and CC: LA (e.g., “I feel that I have too few options to consider leaving this organization”). The authors found that employees’ TIN was significantly higher for the CC: LA-dominant profile than employees with a CC: HS-dominant profile. This finding was also consistent with previous research on CPs (Stanley et al., 2013). That is, if employees with a CC-Dominant profile value the benefit received from their organization in terms of economic value, they are more likely to remain with the organization. On the other hand, if they experience a lack of alternatives, they are more likely to intend to quit. If this is correct, employees from the current study sample who fit Profile 1 (CC-dominant) may experience a lack of alternatives, thus wanting to leave their organizations.

Overall, the results indicated that the nature of mindsets for these two profiles are qualitatively different. Furthermore, the simple dichotomy of moral imperative or indebted obligation is inadequate for explaining the nature of NC in terms of employee mindsets associated with different profiles. The interaction of the commitment components may be more complex than the dual nature of the NC proposition suggests.

### Managerial Implications

This study suggests that senior management should seek ways to implement strategies stemming from CP evaluations to implement training and development initiatives aimed at enhancing employees’ AC. CPs associated with high AC obviously suggest that AC is beneficial to an organization. Then, what are the implications for senior management? It is widely acknowledged that relational leadership practices have been linked to employee retention. When managers regularly interact with employees to strengthen the relationship with their staff and share organizational values to ensure that employees feel comfortable at the workplace, AC is more likely to develop (Rhoades et al., 2001; Meyer et al., 2018).

### Limitations and Future Research

Even though the results demonstrate the necessity of examining profiles of commitment in commitment research, the current study is not without limitations. Namely, the exploratory nature of this study. By adding clarification as to how the three components may amalgamate to produce discrete profiles of commitment regarding TIN and OCB of employees, among other work-related behaviors, the quantitative results of this study allow deeper insight into OC. However, the potential differences between the mindsets associated with each profile cannot be examined based on quantitative results. Therefore, future investigation of CPs should employ qualitative research methods to clarify qualitative employee mindsets associated with CPs. The second limitation is that as the data used in this study is drawn from a larger research project, other variables such as control, predictor, and correlation were not evaluated. Importantly, the lack of research on CPs in non-Western contexts calls for a program of research to demonstrate the construct validity of the profiles and evidence of consistency over time or across non-Western contexts (Morin et al., 2016; Meyer et al., 2018). Thus, future research on CPs should be carried out based on a range of samples to investigate whether the pattern of CPs and results are the same. The third limitation is that this study only conjectured that employees in Profile 1 (CC-Dominant) might experience a lack of alternatives, and did not dichotomize CC when measuring CPs. Thus, future research on CPs should also examine the dual nature of CC.

## Data Availability

All datasets generated for this study are included in the manuscript and/or the supplementary files.

## Ethics Statement

It should be noted that for the theoretical background the author has used some part of his Ph.D. thesis, completed at The Queensland University of Technology under the supervision of Bernd Irmer. However, the rest of the research documented in this paper is new and has never been published elsewhere. The data was collected in South Korea, where non-sensitive data collection (such as, a typical survey of general working adults) is not required for ethical approval for university researchers.

## Author Contributions

The author designed and carried out the experiments, analyzed the data, and wrote the manuscript.

## Conflict of Interest Statement

The author declares that the research was conducted in the absence of any commercial or financial relationships that could be construed as a potential conflict of interest.
